# Incidence and Risk Factors for Respiratory Syncytial Virus and Human Metapneumovirus Infections among Children in the Remote Highlands of Peru

**DOI:** 10.1371/journal.pone.0130233

**Published:** 2015-06-24

**Authors:** Andrew Wu, Philip J. Budge, John Williams, Marie R. Griffin, Kathryn M. Edwards, Monika Johnson, Yuwei Zhu, Stella Hartinger, Hector Verastegui, Ana I. Gil, Claudio F. Lanata, Carlos G. Grijalva

**Affiliations:** 1 School of Medicine, Vanderbilt University, Nashville, Tennessee, United States of America; 2 Division of Infectious Diseases, Washington University School of Medicine, St. Louis, Missouri, United States of America; 3 Department of Pediatrics, Vanderbilt University, Nashville, Tennessee, United States of America; 4 Department of Pathology, Microbiology, and Immunology, Vanderbilt University, Nashville, Tennessee, United States of America; 5 Department of Health Policy, Vanderbilt University, Nashville, Tennessee, United States of America; 6 Vanderbilt Vaccine Research Program, Division of Infectious Diseases, Department of Pediatrics, Vanderbilt University, Nashville, Tennessee, United States of America; 7 Department of Biostatistics, Vanderbilt University, Nashville, Tennessee, United States of America; 8 Swiss Tropical and Public Health Institute, Basel, Switzerland; 9 Instituto de Investigación Nutricional, Lima, Peru; University Hospital San Giovanni Battista di Torino, ITALY

## Abstract

**Introduction:**

The disease burden and risk factors for respiratory syncytial virus (RSV) and human metapneumovirus (MPV) infections among children living in remote, rural areas remain unclear.

**Materials and Methods:**

We conducted a prospective, household-based cohort study of children aged <3 years living in remote rural highland communities in San Marcos, Cajamarca, Peru. Acute respiratory illnesses (ARI), including lower respiratory tract infection (LRTI), were monitored through weekly household visits from March 2009 through September 2011. Nasal swabs collected during ARI/LRTI were tested for RSV, MPV, and other respiratory viruses using real-time RT-PCR. Incidence rates and rate ratios were calculated using mixed effects Poisson regression.

**Results:**

Among 892 enrolled children, incidence rates of RSV and MPV ARI were 30 and 17 episodes per 100 child-years, respectively. The proportions of RSV and MPV ARI that presented as LRTI were 12.5% and 8.9%, respectively. Clinic visits for ARI and hospitalizations were significantly more frequent (all p values <0.05) among children with RSV (clinic 41% and hospital 5.3%) and MPV ARI (38% and 3.5%) when compared with other viral infections (23% and 0.7%) and infections without virus detected (24% and 0.6%). In multivariable analysis, risk factors for RSV detection included younger age (RR 1.02, 95% CI: 1.00-1.03), the presence of a smoker in the house (RR 1.63, 95% CI: 1.12-2.38), residing at higher altitudes (RR 1.93, 95% CI: 1.25-3.00 for 2^nd^ compared to 1^st^ quartile residents; RR 1.98, 95% CI: 1.26-3.13 for 3^rd^ compared to 1^st^ quartile residents). Having an unemployed household head was significantly associated with MPV risk (RR 2.11, 95% CI: 1.12-4.01).

**Conclusion:**

In rural high altitude communities in Peru, childhood ARI due to RSV or MPV were common and associated with higher morbidity than ARI due to other viruses or with no viral detections. The risk factors identified in this study may be considered for interventional studies to control infections by these viruses among young children from developing countries.

## Introduction

Lower respiratory tract infection (LRTI) is the leading global cause of death in children between one month and five years of age [1–4]. Approximately 70% of LRTI deaths under five years of age are among children in developing regions [1, 3]. However, the precise reasons for this disparity in childhood mortality between developed and developing countries are not well understood.

Viruses are commonly detected in childhood respiratory disease in both developed and developing countries [4–8]. Among respiratory viruses, respiratory syncytial virus (RSV) [4–6, 9–22] and human metapneumovirus (MPV) [13, 20–23] are the leading causes of LRTI among children. Although the epidemiology of these viruses has been well characterized in developed countries [24, 25], fewer data exist about their burden and risk factors for developing countries, where their disease burden and related-mortality seem to be the highest [1, 3, 11, 26]. The dearth of information is especially prominent for rural communities where research is typically difficult to conduct due to unique settings, such as high altitudes, that may also influence disease burden [27–29].

Most of the current literature on childhood respiratory disease in developing countries focuses on populations from tropical climates [30], urban areas, or settings where healthcare access is available and information is gathered during healthcare visits [9, 13, 14, 18, 21–24, 30–33]. In rural communities with limited access to health centers, passive surveillance performed at healthcare centers may not reflect the overall disease burden, severity, or risk factors in the community [10, 34, 35]. In those settings, active household-based studies conducted within the community can provide a more comprehensive perspective on disease patterns. Our study sought to quantify the RSV- and MPV-specific burden of acute respiratory infections and to identify risk factors for these infections among children from rural communities in the remote highlands of Peru.

## Materials and Methods

The present study was nested within the RESPIRA-PERU study [36], a prospective, household-based, active surveillance cohort study conducted in the rural highlands of San Marcos, Cajamarca, Peru during March 2009 –September 2011. This region is notably high altitude (1500–4000 meters above sea level) and remote with a population density of 38 persons/km^2^.

### Ethics Statement

Written informed consent was obtained from all participants’ parents or guardians prior to enrollment. The RESPIRA-PERU project was approved by the IRB of both Vanderbilt University and the Instituto de Investigación Nutricional in Peru.

### Study Population

Enrollment criteria have been previously described [37]. Briefly, the study population included children aged <3 years old whose families planned to remain in the study area for at least a year after enrollment.

### The integrating home-based environmental interventions (IHIP) trial

Before the RESPIRA-PERU study, the IHIP trial, a clustered randomized controlled study, was conducted in the same study area [38]. The IHIP trial randomized children to improved cook stoves, solar water disinfection units, and kitchen sinks with running water in order to decrease the frequency of diarrhea and LRTI. Control families were given a mother-child psychomotor stimulation intervention. Children who had participated in the IHIP trial for either arm were eligible to participate in the RESPIRA-PERU study if they met our enrollment selection criteria.

### Household Visits and Data Collection

Data were gathered by local field workers who were trained by study personnel according to the World Health Organization (WHO) Integrated Management of Childhood Illness (IMCI) protocol [39–41]. Field workers visited study participants weekly at their homes to obtain information about any respiratory symptoms during the previous seven days. If one visit was missed, during the next visit information was recorded for the previous 14 days. If two or more consecutive visits were missed, data were recorded only for the 14 days prior to the successful visit.

### Definitions of ARI and LRTI

ARI was defined as the presence of reported fever or cough; and LRTI as the presence of at least one of the following: age-specific tachypnea, audible wheezing, intercostal and subcostal retractions, nasal flaring, or stridor [40]. To distinguish separate episodes of ARI, at least seven days without fever or cough were required between episodes. Only children with ARI symptoms on the day of the visit or the day prior were further evaluated for signs of LRTI as previously defined.

### Collection and analysis of specimens

When an ARI was reported within the previous seven days including the visit day, field workers collected a nasal swab, placed it into Remel M4RT viral transport media (Thermo Fisher Scientific Remel Products, Lenexa, KS) and delivered to the local research laboratory in San Marcos within 8 hours of collection for processing and storage. Samples were subsequently shipped to Vanderbilt University for testing using real-time RT-PCR targeting the following respiratory viruses: rhinovirus, adenovirus, parainfluenza (types 1, 2, 3), influenza (A, B, C), RSV and MPV. [36].

### RSV and MPV Seasons

To assure that our risk factor assessment was restricted to periods at risk for RSV and MPV infections, risk factor analyses were only performed during seasons when RSV and MPV were circulating. The beginning of a specific season was defined as the first week of continuous RSV/MPV activity with one or more test positives in two consecutive weeks. The end of a season was defined as the last week of two consecutive weeks with at least one infection per week.

### Statistical Analysis

Descriptive analysis was conducted and frequencies, means, medians, and proportions were reported for RSV episodes without MPV co-detection and for MPV episodes without RSV co-detection. Wilcoxon rank sum tests for continuous data and Chi-squared tests for contingency tables were used to examine differences in clinical attributes between RSV and MPV episodes in which only one virus was detected. For comparison purposes, we grouped non-study viruses as “Other Virus” encompassing rhinovirus, adenovirus, parainfluenza (types 1, 2, 3), or influenza (A, B, C) infections. A separate “No Virus Detected” group included those ARI in which none of the tested viruses were detected.

Incidence rates were calculated by dividing the number of new ARI’s by person-time of observation at risk with the rates expressed as ARI incidence per 100 child-years, while accounting for individual and village clustering to obtain robust standard errors, and 95% CI’s of ARI incidence rates were reported. Incidence rate comparisons were performed using incidence rate ratios. For the identification of risk factors, we fit virus-specific multivariable mixed effects Poisson regression models. Factors evaluated simultaneously included age, gender, calendar year, sharing a bed, the number of household members per bedroom, day care attendance, presence of a smoker, maternal education level, dirt flooring, the household head’s occupation, presence of electricity, water source, disposal services and sewage, altitude, and IHIP participation status. Multilevel multiple imputation was performed to account for observations with missing covariate data (<8%) [42]. Analyses were repeated in a complete case scenario (without data imputation). All statistical analyses were performed using Stata 13.1 software (STATACorp College Station, TX).

## Results

The RESPIRA-PERU study enrolled 892 children from 810 households in 58 communities in the San Marcos province. There were 4,475 ARI episodes during a total of 755.1 child-years at risk (median follow-up of 0.81 child-years per child), with 226 episodes involving RSV and 130 involving MPV. Co-infection with RSV and MPV occurred in only two ARI episodes. 202 (23%) children had at least 1 RSV infection and 127 (14%) children had at least 1 MPV infection. Of note, in 42% of RSV ARI and 33% of MPV ARI other study viruses were co-detected, with rhinovirus responsible for 75% of the RSV co-detections and 65% of the MPV co-detections.

The average age of participants across all infection categories was 15 months. Approximately half of all participants were female. Most participants had dirt floors in their houses (>91%), used a solid form (coal or wood) of cooking fuel (>91%), lived with a household member who worked in agriculture (>76%), and had mothers who did not complete secondary school (>84%) [36]. These characteristics reflect the rural setting and poverty in which these participants reside. Additionally, 3 out of 4 participants across all infection categories resided at altitudes higher than 2314 meters above sea levels. Demographics and characteristics associated with infections involving RSV, MPV, other viruses, and no viral detections are shown in Table 1.

**Table 1:** Demographics and characteristics associated with ARI episodes by virus type.

Characteristic	RSV (n = 224)	MPV (n = 128)	Other Viral (n = 2294)	No Virus Detected (n = 1309)
Age in months, mean (95% CI)	15.4 (14.1–16.7)	15.9 (14.2–17.6)	15.9 (15.5–16.3)	14.1 (13.6–14.7)
Female	49.5 (44–57)	53.9 (47.4–55)	47.8 (46.1–59.2)	47.3 (45–50.4)
Year				
2009	10.7 (6.7–14.8)	9.4 (4.3–14.4)	19.7 (18.1–21.3)	26.4 (24–28.8)
2010	41.5 (35–48)	27.3 (19.6–35.1)	49.8 (47.7–51.8)	48.4 (45.7–51.1)
2011	47.8 (41.2–54.3)	63.4 (54.9–71.7)	30.5 (28.6–32.4)	25.1 (22.8–27.5)
Shared a bed	98.2 (96.4–99.9)	99.2 (97.7–1.01)	97.6 (96.9–98.2)	96.9 (96–97.9)
Attended day care	7.2 (3.8–10.6)	5.5 (1.5–9.4)	7.4 (6.3–8.5)	6.3 (5–7.6)
People per household, median (IQR)	5 (4–7)	5 (4–6)	5 (4–6)	5 (4–6)
Children <5y per household, median (IQR)	1 (1–2)	1 (1–2)	1 (1–2)	1 (1–2)
Number of bedrooms, median (IQR)	2 (1–2)	1 (1–2)	1 (1–2)	1 (1–2)
Electricity in home	40.2 (33.7–46.6)	48.4 (39.7–57.1)	40.7 (38.7–42.7)	43.2 (40.5–45.9)
Water from pipeline or well	80.4 (75.1–85.6)	88.3 (82.7–93.9)	85.3 (83.8–86.7)	86.6 (84.8–88.5)
Municipal sewer or septic tank	21 (15.6–26.3)	25 (17.5–32.5)	20 (18.4–21.7)	19.1 (17–21.3)
Dirt floor	93.8 (90.6–96.9)	85.9 (80–92)	91.2 (90–92.4)	89.9 (88.3–91.5)
Cooking fuel				
Gas	4.0 (1.5–6.6)	3.1 (0.1–6.1)	3.7 (2.9–4.4)	4.8 (3.7–6.0)
Wood	94.6 (91.7–97.6)	96.1 (92.7–99.5)	95.2 (94.3–96.1)	93.4 (92–94.7)
Head of household in agriculture	77.7 (72.2–83.1)	68 (60–76.1)	76.7 (75–78.5)	76.1 (73.7–78.4)
Mother did not complete secondary school	87.7 (83.4–92.1)	85.8 (79.7–91.9)	88.9 (87.6–90.2)	87.1 (85.3–89)
Altitude quartile (m)				
1976–2314	17.4 (12.4–22.4)	26.6 (18.9–34.2)	25.4 (23.6–27.2)	26.4 (24–28.7)
2315–2626	27.7 (21.8–33.6)	25.8 (18.2–33.4)	24.5 (22.8–26.3)	24.5 (22.2–26.9)
2628–2865	27.7 (21.8–33.6)	19.5 (12.6–26.4)	24.9 (23.2–26.7)	26.3 (23.9–28.7)
2866–3803	27.2 (21.4–33.1)	28.1 (20.3–35.9)	25.2 (23.4–26.9)	22.8 (20.6–25.1)
IHIP trial status				
Non-participant	85.7 (81.1–90.3)	88.3 (82.7–93.9)	81.2 (79.6–82.8)	81.5 (79.4–83.6)
Participant, control arm	6.3 (3.1–9.4)	7 (2.6–11.5)	11.2 (9.9–12.4)	9.4 (7.8–10.9)
Participant, intervention arm	8 (4.5–11.6)	4.7 (1–8.4)	7.6 (6.5–8.7)	9.1 (7.5–10.6)

Data are displayed as percentages (95% CI) unless otherwise noted.

RSV and MPV values were calculated using infections either with RSV + no MPV or MPV + no RSV. Therefore, this data do not necessarily reflect isolated infections. The two RSV+MPV episodes were not included for this table. 42% of RSV ARI and 33% of MPV ARI were co-detected with other study viruses. “Other Viral” ARI included rhinovirus, adenovirus, parainfluenza (types 1, 2, 3), and influenza (A, B, C).

Although the mean age for both infections was similar (16 months), the risk of RSV and MPV infections differed by age. The incidence of RSV was highest in children under 12 months of age (34 per 100 child-years, 95% CI: 25–46) and lowest in children from 24–35 months (25, 95% CI: 19–32). In contrast, the highest rate of MPV infection was in children between 6–11 months (27, 95% CI: 19–37) and lowest in children under 6 months of age (12, 95% CI: 14–20). The RSV incidence rates were significantly higher than MPV rates for each age group except among children 6–11 months of age (p = 0.14) (Table 2).

**Table 2:** RSV and MPV incidence rates per 100 child-years stratified by age.

Viral Infection Type	Total Cases	Total Child-years	Incidence rate per 100 child-years (95% CI)
RSV			
All ages (months)	226	755	30 (26–34)
0–5	44	128	34 (25–46)
6–11	47	139	34 (25–45)
12–23	77	253	31 (24–38)
24–35	58	235	25 (19–32)
MPV			
All ages	130	755	17 (14–20)
0–5	16	128	12 (8–20)
6–11	37	139	27 (19–37)
12–23	39	253	15 (11–21)
24–35	38	235	16 (12–22)

Table 3 describes the clinical manifestations of RSV and MPV infections. The reported days of fever among RSV and MPV infections were each longer than duration of fever in infections where no virus was detected. RSV ARI was more likely to present as an LRTI and to result in an ARI clinic visit or hospitalization compared with ARI due to other viral infections and ARI in which no virus was detected. Similarly, MPV infections were more likely to present with tachypnea and result in an ARI clinic visit or hospitalization when compared to other viral ARI and ARI with no virus detected. RSV and MPV accounted for approximately 30.4% and 13% of virus-positive hospitalizations where one virus was detected. The next most common virus–rhinovirus–was detected in approximately 21.8% of hospitalizations. Approximately 10.7% and 6.9% of RSV and MPV ARI were associated with LRTIs, yielding incidence rates of 1.8 and 0.8 LRTIs per 100 child-years, respectively. The clinical manifestations of the ARI with a single virus were not significantly different from ARI that included other viral co-detections.

**Table 3:** Clinical attributes by viral infection type.

Viral Infection	Age in months, mean	Symptom Duration (days)	Fever (days)	Cough (days)	Rhinorrhea (days)	Audible Wheezingθ (%)	Tachypneaθ (%)	Any sign of LRTIθ (%)	Clinic visit for ARI (%)	Hospitalized (%)
RSV* (n = 131)	16.1 (14.3–17.9)	8 (5–10)	2 (1–3)	7 (5–10)	6 (4–8)	1.5 (0–3.6)	9.2 (4.2–14.1)	10.7 (5.4–16)	41 (32.8–50)	5.3 (1.5–9.2)
RSV** (n = 224)	15.4 (14.1–16.7)	7 (5–11)	2 (0–3)	6 (4–10)	6 (3–9)	1.3 (0–2.8)	9.4 (5.6–13.2)	10.7 (6.7–14.8)	40.6 (23.2–47.1)	4.0 (1.4–6.6)
MPV* (n = 87)	15.9 (13.8–18.1)	7 (5–10)	2 (1–4)	6 (4–9)	6 (3–9)	1.1 (0–3.4)	6.9 (1.6–12.2)	6.9 (1.6–12.2)	38 (27.7–48.1)	3.5 (0–7.3)
MPV** (n = 128)	15.9 (14.2–17.6)	7 (4–9)	2 (1–4)	6 (4–9)	6 (3–8)	0.8 (0–2.3)	7.8 (3.2–12.5)	7.8 (3.2–12.5)	35 (26.9–43.4)	2.3 (0–5)
Other Virus* (n = 1885)	15.5 (15.1–16)	5 (3–9)	1 (0–2)	5 (2–8)	4 (2–7)	0.3 (0.1–0.6)†	2.4 (1.7–3.1)†ⱡ	3.7 (2.8–4.5)†	23 (21–25)†ⱡ	0.7 (0.3–1.1)†ⱡ
No Virus Detected	14.1 (13.6–14.7)	4 (2–8)ⱡ	2 (1–3)†ⱡ	3 (0–6)	2 (0–5)	0.5 (0.1–0.9)	1.2 (0.6–1.8)†ⱡ	2.4 (1.6–3.3)†ⱡ	24 (21.8–26.4)†ⱡ	0.6 (0.2–1.0)†ⱡ

Data represent median values (IQR) and proportions (95% CI).

Values that were significantly different (p<0.05) when compared to RSV* (†) and MPV* (ⱡ).

*These values for RSV, MPV, and other virus infections include infections with only 1 virus detected.

**These values include co-infections.

θWheezing, tachypnea, and any LRTI symptoms were calculated among all children with ARI regardless of whether or not an exam was performed for the given symptom. Tachypnea was defined separately for specific age groups.

The risk factors for RSV and MPV infections also differed. In multivariable analyses younger age, the presence of a smoker, and living at higher altitudes (specifically 2315–2865 meters above sea level compared to 1976–2314 meters) were identified as independent risk factors for RSV infection. The only significant risk factor for MPV in multivariable analysis was the occupation of the household head, which was not so for RSV. Specifically, higher MPV risk was associated with unemployed household heads when compared with household heads with some type of employment (Table 4). Separate analyses without data imputation yielded results similar to those from the main analyses (S1 Table).

**Table 4:** Risk factors for specific viral infections.

	RSV (n = 184)				MPV (n = 102)			
	Univariate		Multivariable		Univariate		Multivariable	
Characteristic	Rate Ratio (RR) (CI)	P value	RR (CI)	P value	RR (CI)	P value	RR (CI)	P value
Age (months)	0.98 (0.97–1.00) †	0.01	0.98 (0.96–0.99) †	0.01	0.99 (0.97–1.01)	0.24	1.00 (0.98–1.03)	0.77
Female	0.92 (0.70–1.21)	0.56	0.95 (0.73–1.25)	0.73	1.06 (0.74–1.52)	0.74	1.12 (0.79–1.60)	0.53
Year								
2009	Reference							
2010	0.85 (0.48–1.52)	0.58	0.83 (0.44–1.56)	0.57	0.91 (0.38–2.18)	0.83	0.64 (0.21–1.91)	0.43
2011	1.00 (0.57–1.77)	0.99	1.05 (0.53–2.09)	0.90	1.41 (0.63–3.15)	0.41	0.78 (0.24–2.48)	0.67
Shares a bed	2.72 (0.73–10.2)	0.14	2.53 (0.66–9.70)	0.18	2.73 (0.41–18.2)	0.30	3.23 (0.51–20.5)	0.21
Persons per bedroom	0.99 (0.95–1.04)	0.79	0.99 (0.93–1.04)	0.61	0.96 (0.88–1.04)	0.31	0.97 (0.90–1.05)	0.49
Attended day care	1.01 (0.62–1.64)	0.97	1.33 (0.80–2.21)	0.28	0.70 (0.31–1.60)	0.40	0.78 (0.35–1.76)	0.55
Smoker in the house	1.62 (1.13–2.33) †	0.01	1.63 (1.12–2.38) †	0.01	1.45 (0.88–2.37)	0.14	1.42 (0.84–2.38)	0.19
Dirt floor	1.50 (0.88–2.54)	0.14	1.51 (0.87–2.61)	0.14	0.67 (0.40–1.11)	0.12	0.75 (0.44–1.29)	0.30
Electricity in home	0.86 (0.65–1.14)	0.30	1.05 (0.78–1.42)	0.73	1.22 (0.85–1.75)	0.27	1.04 (0.69–1.58)	0.84
Water from pipeline or well	0.78 (0.55–1.11)	0.17	0.81 (0.55–1.19)	0.28	1.72 (0.88–3.37)	0.11	1.71 (0.87–3.34)	0.12
Municipal sewer or septic tank	0.92 (0.66–1.28)	0.61	1.02 (0.72–1.45)	0.90	1.05 (0.68–1.63)	0.87	0.87 (0.53–1.42)	0.57
Occupation of household head								
Non-agricultural	Reference							
Agricultural	1.03 (0.72–1.48)	0.86	0.80 (0.55–1.63)	0.24	0.71 (0.46–1.08)	0.11	0.68 (0.42–1.08)	0.10
Unemployed/other	0.70 (0.28–1.76)	0.45	0.65 (0.24–1.76)	0.40	2.19 (1.19–4.03) †	0.01	2.11 (1.12–4.01) †	0.02
Mother did not complete secondary school	1.32 (0.85–2.05)	0.22	1.13 (0.74–1.74)	0.58	0.97 (0.59–1.59)	0.90	1.25 (0.74–2.11)	0.40
Altitude quartile (m)								
1976–2314	Reference							
2315–2626	2.06 (1.34–3.17) †	<0.01	1.93 (1.25–3.00) †	<0.01	1.17 (0.74–1.84)	0.51	1.29 (0.83–2.01)	0.26
2628–2865	1.82 (1.18–2.82) †	0.01	1.98 (1.26–3.13) †	<0.01	0.62 (0.36–1.09)	0.10	0.83 (0.46–1.50)	0.54
2866–3803	1.75 (1.10–2.76) †	0.02	1.67 (0.99–2.79)	0.05	0.91 (0.55–1.50)	0.70	1.21 (0.70–2.09)	0.49
IHIP trial status								
Non-participant	Reference							
Participant, control	0.56 (0.30–1.02)	0.06	0.78 (0.39–1.56)	0.48	0.70 (0.35–1.41)	0.32	0.68 (0.25–1.83)	0.44
Participant, intervention	0.84 (0.53–1.32)	0.45	1.09 (0.60–1.99)	0.78	0.39 (0.15–1.04)	0.06	0.42 (0.13–1.36)	0.15

Values that were significantly different (p<0.05) when compared to reference values are indicated by the symbol †. Numbers of RSV and MPV infections are smaller than Table 2 because analysis was restricted to infections occurring during the RSV or MPV season among children who were observed and at risk during each season.

## Discussion

In our study, ARI due to RSV and MPV were very common among young children living in rural communities of the Peruvian Andes. RSV and MPV ARI were also more likely to exhibit indicators of severe disease such as tachypnea, clinic visits, and hospitalizations than ARI from other viral causes or when no viruses were detected. Risk factors for RSV detection were younger age, presence of an indoor smoker, and living at high altitudes. Conversely, age, smoking, and altitude were not associated with MPV infection risk. The only significant risk factor for MPV ARI was the head of household’s occupation, with higher risk associated with unemployment.

Our RSV ARI incidence rate of 30 events per 100 child-years is similar to that of prospective, household-based studies conducted in two low resource settings at lower altitude (about 36 events per 100 child-years) [17, 30]. To our knowledge, the only other household-based study that has assessed the burden of MPV infections in young children found an incidence rate of 7 per 100 child-years among the urban poor of Bangladesh [21], which was much lower than our observed MPV rate of 17 per 100 child-years. Of note, that study in Bangladesh required one major or multiple minor criteria for physician referral where the final ARI diagnosis was made, which may explain this difference in incidence rates. In previous studies performed by our group among young U.S. children, the rates of outpatient clinic visits for RSV and MPV infections in infants were much higher than the rates in this household-based study [24, 25]. This may largely be a reflection of greater access to outpatient care.

The age distribution of RSV and MPV appeared to have distinct patterns. RSV incidence rates were highest in children less than 12 months whereas MPV rates were highest only in the 6–11 month age range. Consistent with our results, several studies conducted at health facilities in developed countries have reported that MPV tend to affect older infants whereas RSV affects both younger and older infants [13, 24]. For example, rates of RSV among outpatient children <6 months and 6–11 months were both high and similar to each other in a study conducted by our colleagues among US children [25]. In another study by our group, rates of MPV were higher for those 6–11 months than less than 6 months in that same population of US children [24]. Similar RSV and MPV studies conducted in developing regions only report LRTI rates, but demonstrate similar age patterns, with RSV LRTI incidence rates highest among infants 0–6 months and MPV LRTI rates highest in 6–12 month olds [21, 43]. Although these studies have been conducted in different settings and use different methodologies, it appears that the age distribution of RSV and MPV ARI may be similar between studies conducted in developing and developed regions.

There was substantial clinical overlap between RSV and MPV ARI regarding duration of different symptoms. This is not surprising since both viruses belong to the same viral subfamily, tend to circulate between December and April in the Northern hemisphere in developed countries [23, 24, 32], and both are associated with bronchiolitis [4, 13, 22, 23]. Furthermore, these viruses frequently co-circulate during respiratory seasons in our study region as well [36]. These two viruses have similar patterns of severity in our study. The overlap of certain clinical manifestations such as audible wheezing and tachypnea seen with bronchiolitis highlights the frequent involvement of the lower respiratory tract with these two viruses. Other studies in developing regions often cite RSV and MPV as the most common causes of LRTI compared to other common childhood respiratory pathogens [10, 20–23]. Of note, some of those studies defined LRTI as hospitalization or presence of tachypnea alone. Therefore, our results seem consistent with those studies since RSV and MPV did have significantly stronger associations with hospitalization compared to non-RSV and non-MPV infections. Our percentages of virus-positive hospitalizations attributable to RSV and MPV were also consistent with similar studies. LRTI among hospitalized children in Brazil were associated with RSV and MPV in 86% of all the virus-positive cases, while the other viruses comprised 14% of episodes [20]. Other studies by our group in US health facilities found that 20% and 6% of children under 5 years who were hospitalized for ARI were due to RSV and MPV, respectively [24, 25]. Overall, it appears that RSV and MPV ARI are significantly associated with indicators of more severe disease in both developed and developing countries.

Previous studies in low-resource settings have found that crowding is a significant risk factor for severe childhood RSV infection [14, 30, 33, 44, 45]. In our study, RSV and MPV ARI were not significantly associated with crowding. This finding should be interpreted with caution. First, crowding likely presents more risk indoors than outdoors and our study is unable to differentiate between times spent in these two settings. Additionally, the number of persons per household in our study (median of 5) was low compared to similar studies that did detect an association with crowding. For example, a study conducted in rural Alaska [33], where it is common to have 6 people per household [44], found a significant relationship between crowding and RSV hospitalizations of children >6 months only when the number of people in the household was at least eight. Similarly, a study in Gambia [14] used a threshold of 10 household members to define crowding. It should be noted that these studies assessed for hospitalization as a primary outcome, whereas we focused on all respiratory disease episodes. The risk factor of crowding may be limited to RSV hospitalization risk. Consistent with this observation is the finding from a Kenyan study [30] that increasing numbers of children in the household were not associated with RSV ARI, but was associated with LRTI when the number of children exceeded ten. Unfortunately, evaluation of the relationship between hospitalizations and crowding was unable to be performed in our study due to the low number of hospitalizations from these rural isolated communities.

A number of studies conducted in developed [13, 18] and developing countries [31] have reported that having a smoker in the house increases the risk of RSV infection. Okiro et al [30] found that RSV ARI risk increased significantly if at least 2 smokers were present in the household. Other studies have specifically demonstrated an association between maternal smoking and LRTI in the first year of life [46, 47]. In our study, we observed a significant relationship between indoor smoking and RSV infection but the association with MPV infection did not reach statistical significance. Although the point estimates were similar to associations previously reported, the prevalence of smoking was relatively low in the study population and the precision of our MPV estimates was limited. Interestingly, we also noted that although the risk of RSV infections was not strongly associated with the household head’s occupation, we observed a significant association between a MPV infection rate and household head’s occupation. This observation is intriguing but requires subsequent assessment in other settings.

A previous article on risk factors for RSV ARI reported that children living above 2500 meters were 33% more likely to be hospitalized for RSV compared to those living below 2500 meters [29]. Similarly, a 2008 WHO report mentions “high altitude (cold air)” as a possible risk factor for childhood community-acquired pneumonia [28]. This is congruent with our results, which show that the risk of RSV ARI increases with increasing altitude beyond 2300 meters. Besides the temperature differences, higher altitudes generally have lower oxygen saturation levels, can induce pulmonary vasoconstriction secondary to hypoxia, and can cause impaired nasal mucociliary transport, all of which may affect susceptibility to respiratory infection [27, 48]. In our study, however MPV infection rates appear to be less affected by altitude, which may suggest unique aspects of MPV infection, such as the effects of temperature on viral replication, transmission, or virulence. To our knowledge, this is one of few studies that have specifically explored the association of MPV infection with high altitude in the literature.

Our findings must be interpreted in light of some limitations. Our risk factor analyses required restrictions such as stratifying the primary participant group that likely limited our sample size and statistical power to detect some associations. Second, other relevant variables such as time-varying nutritional or immunological status of the study children were not gathered as part of the study, and thus remained unaccounted for in our analyses. Third, samples were only gathered during ARI episodes, though virus could have been present during asymptomatic periods. Nevertheless, previous studies using molecular detection techniques have consistently shown that RSV and MPV were rarely detectable in asymptomatic young children [32, 49, 50], which should mitigate that concern. Fourth, although viruses account for a majority of respiratory disease in children, bacteria also play an important role in severe disease. The bacterial etiology of study ARIs was not assessed in this study. In a number of RSV and MPV infections we also detected other viruses, especially rhinovirus. Although these co-infections might impact disease severity, the majority of RSV and MPV infections in our study did not have viral co-detections and we did not find significant differences in clinical manifestations or healthcare use between those with and without co-detections. Finally, even though our study was conducted in the rural, highland communities of the Andes, these results may not represent other populations who also live in high altitudes. Nevertheless, these populations are typically excluded from traditional research, and information on the epidemiology of viral infections in this setting is lacking.

In conclusion, RSV and MPV ARI were very common among young Andean children. Both viruses shared many clinical symptoms between each other as well as with other viruses. RSV and MPV ARI notably presented with more indicators of severe disease than infections due to other viral causes. The risk factors identified in this study may be considered for further interventions to control infections by these viruses among young children from developing countries.

## Supporting Information

S1 Table Risk factors for specific viral infections without imputation. Values that were significantly different (p<0.05) when compared to reference values are indicated by the symbol †. Numbers of RSV and MPV infections are smaller than Table 3 because analysis was restricted to infections occurring during the RSV or MPV season among children who were observed and at risk during each season.
(DOCX)
