# Supplementary material for: Incidence and Risk Factors for Respiratory Syncytial Virus and Human Metapneumovirus Infections among Children in the Remote Highlands of Peru
Source: PLoS One. 2015 Jun 24;10(6):e0130233. doi: 10.1371/journal.pone.0130233 (PMC4479592; doi:10.1371/journal.pone.0130233)
Supplement: S1 Table — Values that were significantly different (p<0.05) when compared to reference values are indicated by the symbol †. Numbers of RSV and MPV infections are smaller than Table 3 because analysis was restricted to infections occurring during the RSV or MPV season among children who were observed and at risk during each season. (DOCX) [file pone.0130233.s001.docx]

|  | RSV (N=184) | | | | MPV (N=102) | | | |
| --- | --- | --- | --- | --- | --- | --- | --- | --- |
|  | Univariate | | Multivariable | | Univariate | | Multivariable | |
| Characteristic | Rate Ratio (RR) (CI) | P value | RR (CI) | P value | RR (CI) | P value | RR (CI) | P value |
| Age (months) | 0.98 (0.97-1.00) † | 0.02 | 0.97 (0.96-0.99) † | 0.01 | 0.99 (0.97-1.01) | 0.27 | 1.00 (0.98-1.03) | 0.88 |
| Female | 0.92 (0.69-1.23) | 0.59 | 0.99 (0.73-1.34) | 0.95 | 1.06 (0.72-1.57) | 0.76 | 1.12 (0.75-1.67) | 0.57 |
| Year |  |  |  |  |  |  |  |  |
| 2009 | Reference | | | | | | | |
| 2010 | 0.85 (0.46-1.56) | 0.60 | 0.84 (0.36-1.93) | 0.68 | 0.91 (0.37-2.21) | 0.83 | 0.44 (0.15-1.26) | 0.13 |
| 2011 | 1.00 (0.55-1.82) | 0.99 | 1.05 (0.43-2.54) | 0.92 | 1.41 (0.61-3.24) | 0.42 | 0.54 (0.18-1.62) | 0.27 |
| Shares a bed | 2.70 (0.67-10.9) | 0.16 | 2.46 (0.59-10.2) | 0.22 | 2.71 (0.38-19.4) | 0.32 | 3.33 (0.45-24.8) | 0.24 |
| Persons per bedroom | 0.99 (0.90-1.09) | 0.85 | 0.98 (0.88-1.09) | 0.74 | 0.90 (0.78-1.04) | 0.14 | 0.96 (0.83-1.11) | 0.59 |
| Attended day care | 1.01 (0.57-1.77) | 0.98 | 1.18 (0.62-2.24) | 0.61 | 0.71 (0.29-1.73) | 0.45 | 0.82 (0.32-2.11) | 0.69 |
| Smoker in the house | 1.60 (1.07-2.39) † | 0.02 | 1.59 (1.04-2.42) † | 0.03 | 1.48 (0.86-2.57) | 0.16 | 1.42 (0.80-2.52) | 0.23 |
| Dirt floor | 1.50 (0.83-2.68) | 0.18 | 1.53 (0.80-2.89) | 0.20 | 0.67 (0.37-1.20) | 0.17 | 0.76 (0.39-1.48) | 0.41 |
| Electricity in home | 0.86 (0.64-1.16) | 0.33 | 1.05 (0.74-1.48) | 0.80 | 1.21 (0.82-1.79) | 0.33 | 1.04 (0.65-1.66) | 0.86 |
| Water from pipeline or well | 0.78 (0.54-1.13) | 0.19 | 0.78 (0.52-1.16) | 0.23 | 1.72 (0.90-3.31) | 0.10 | 1.80 (0.88-3.65) | 0.11 |
| Municipal sewer or septic tank | 0.91 (0.63-1.32) | 0.63 | 1.07 (0.72-1.61) | 0.73 | 1.04 (0.64-1.68) | 0.87 | 0.84 (0.49-1.45) | 0.53 |
| Occupation of household head |  |  |  |  |  |  |  |  |
| Non-agricultural | Reference | | | | | | | |
| Agricultural | 1.03 (0.71-1.50) | 0.86 | 0.81 (0.53-1.24) | 0.34 | 0.71 (0.44-1.13) | 0.15 | 0.65 (0.38-1.12) | 0.12 |
| Unemployed/other | 0.70 (0.25-1.98) | 0.50 | 0.71 (0.24-2.07) | 0.53 | 2.18 (1.01-4.72) † | 0.047 | 2.15 (0.92-5.00) | 0.08 |
| Mother did not complete secondary school | 1.30 (0.83-2.03) | 0.25 | 1.14 (0.70-1.85) | 0.60 | 1.00 (0.58-1.72) | 0.99 | 1.25 (0.68-2.29) | 0.47 |
| Altitude quartile (m) |  |  |  |  |  |  |  |  |
| 1976-2314 | Reference | | | | | | | |
| 2315-2626 | 1.85 (1.19-2.87) † | 0.01 | 1.80 (1.12-2.88) † | 0.02 | 1.10 (0.66-1.84) | 0.71 | 1.30 (0.76-2.24) | 0.34 |
| 2628-2865 | 1.71 (1.10-2.67) † | 0.02 | 1.95 (1.20-3.18) † | 0.03 | 0.61 (0.34-1.11) | 0.10 | 0.85 (0.44-1.64) | 0.63 |
| 2866-3803 | 1.66 (1.07-2.59) † | 0.03 | 1.59 (0.94-2.68) | 0.08 | 0.92 (0.54-1.55) | 0.75 | 1.22 (0.64-2.33) | 0.54 |
| IHIP trial status |  |  |  |  |  |  |  |  |
| Non-participant | Reference | | | | | | | |
| Participant, control | 0.56 (0.29-1.06) | 0.07 | 0.86 (0.38-1.99) | 0.73 | 0.70 (0.32-1.50) | 0.36 | 0.75 (0.27-2.08) | 0.58 |
| Participant, intervention | 0.84 (0.50-1.41) | 0.51 | 0.91 (0.42-1.97) | 0.81 | 0.39 (0.14-1.06) | 0.06 | 0.37 (0.10-1.37) | 0.14 |
|  |  |  |  |  |  |  |  |  |
